# Structural impairment and conflict load as vulnerability factors for burnout – A cross-sectional study from the German working population

**DOI:** 10.3389/fpsyg.2022.1000572

**Published:** 2023-01-24

**Authors:** Julia Perlinger, Hannes Gisch, Johannes C. Ehrenthal, Christiane Montag, Thomas Kretschmar

**Affiliations:** ^1^Mind-Institute SE., Berlin, Germany; ^2^Department of Psychology, University of Cologne, Cologne, Germany; ^3^Department of Psychiatry and Neurosciences, CCM, Charité Universitätsmedizin Berlin, Corporate Member of Freie Universität Berlin and Humboldt-Universität zu Berlin, Berlin, Germany

**Keywords:** OPD, structural impairment, conflict modes, coping, burnout

## Abstract

**Introduction:**

Individual vulnerability and resilience factors are increasingly studied in burnout research. This is especially true for clinical variables that translate directly into intervention programs from a psychodynamic perspective. For example, few studies have examined the relationship between structural impairment and the individual spectrum of motivational conflicts according to the Operationalized Psychodynamic Diagnosis system (OPD) in relation to burnout. To substantiate previous findings, we hypothesized that structural impairment as well as motivational conflicts are related to burnout, but that structural impairment explained additional variance and mediated a possible relationship between conflicts and burnout.

**Method:**

The present cross-sectional study was carried out on a sample of the German working population (*N* = 545). Questionnaires were used to measure structural impairment (OPD-SQS), the conflict-modes along with the category K0 (OPD-CQ), as well as burnout (BOSS-I/-II).

**Results:**

Structural impairment, a number of conflict modes, and burnout were significantly associated. Moreover, structural impairment explained additional variance in burnout. The requirements for the conflict-specific mediation models were given for 9 of the 12 OPD conflict modes. In these models the impact of the conflict modes on burnout was mediated by structural impairment.

**Discussion:**

The current study broadens the comprehension of the relations between structural impairment, the conflict modes and burnout. In addition it higlights the role of structural impairment in predicting burnout risk and possible prevention approaches.

## 1. Introduction

Mental disorders and their importance in terms of incapacity to work and early retirement due to illness are discussed from social, political, and scientific perspectives (Ahola et al., 2008). In this context, burnout plays an increasingly significant role (Federal Institute for Occupational Safety and Health, 2018). In general, burnout is defined as an exhaustion syndrome resulting from unresolved, job related chronic stress (Maslach, 2003). The most widely accepted definition of burnout is the one of Maslach et al. (1996). According to them, burnout is composed of the three dimensions of emotional exhaustion, depersonalization and reduced personal accomplishment (Maslach et al., 1996). While emotional exhaustion describes an individual’s lack of physical as well as emotional resources, depersonalization reflects a negative, callous, or detached attitude toward their work and the people they work with. Finally, reduced personal accomplishment represents feelings of incompetence and poor performance at work (Maslach et al., 1996). Additionally, in the ICD-11, which is due to come into force in 2022, burnout is defined by the dimensions “emotional exhaustion and distancing from professional activity,” “negativistic or even cynical attitude toward one’s own profession,” and “reduced job performance” (World Health Organization, 2019). Moreover, burnout is considered a stress-induced risk state for physical as well as psychological secondary diseases (Salvagioni et al., 2017). While the extent to which burnout represents an independent disorder and can be distinguished from depression is still being investigated (Bianchi et al., 2015), its status as a workplace-related risk factor is well documented. However, it is generally accepted that not only environmental influences but also personality factors play a role in the development of burnout (Schaufeli et al., 1993; Salvagioni et al., 2017). Personal characteristics do not only influence how we perceive stressful situations, but also facilitate or impair the way we adapt to them (Kaplan, 1996). In addition to sociodemographic data, such as gender (Purvanova and Muros, 2010), age (Gómez-Urquiza et al., 2017), and level of education (Farshi and Omranzadeh, 2014), several personality traits have previously been associated with burnout. In previous studies self-efficacy, internal locus of control, emotional stability, hardiness, or proactivity have been identified as resources in relation to burnout (Alarcon et al., 2009). Moreover, the big-five personality dimensions of extraversion, openness, agreeableness, and conscientiousness (Swider and Zimmerman, 2010) as well as emotional intelligence (Mérida-López and Extremera, 2017), were found to be protective factors related to burnout. On the other hand, perfectionistic concerns (Hill and Curran, 2015), early maladaptive schemas (Simpson et al., 2019), high novelty seeking (Khosravi et al., 2020), and the big-five dimension neuroticism (Swider and Zimmerman, 2010; Roloff et al., 2022) were identified as vulnerability factors in relation to burnout.

There is much less research on vulnerability factors that relate directly to clinical models of personality and psychopathology from a modern psychodynamic perspective. This, however, is important as some perspectives on treating burnout are related to psychodynamic models (i.e., Malach-Pines and Yafe-Yanai, 2001). A well-established multiaxial diagnostic instrument in German-speaking countries is the Operationalized Psychodynamic Diagnosis system (OPD) (OPD Task Force, 2011). Here, too, the subjective experience and processing capacity of external stress are seen as a consequence of individual personality differences (OPD Task Force, 2011). In this context, structural impairment, or “structure” (Axis-IV) and individual motivational conflicts (Axis-III) play an important role (Rudolf et al., 2004).

According to the OPD, “structure” is defined as the availability of psychological functions for the regulation of the self in its relationship with internal and external objects (OPD Task Force, 2011). The development of personality structure originates in early dyadic relationships and is characterized by the capacity for perception, regulation, communication, and attachment in relation to one’s inner life as well as to other people. Table 1 provides an overview of the dimensions and facets of personality structure (OPD Task Force, 2011). Moreover, the regulation of affects represents the main component of the personality structure (OPD Task Force, 2011). Trauma or deprivation experiences at an early age can result in deficits in the development of personality structure. This, in turn, can lead to a reduced ability to regulate internal and external states of tension, which makes it difficult to cope with developmental tasks and stressful situations later in life (OPD Task Force, 2011; Bugaj et al., 2016). In general, previous research found that people with structural impairments are prone to various mental illnesses (Grande et al., 1998; Mestel et al., 2004; Benecke et al., 2009, 2018; Ehrenthal et al., 2012; Obbarius et al., 2019; Baie et al., 2020; Bröcker et al., 2020). Furthermore, it has been shown that structural impairment is higher in patients with a personality disorder than in patients without a personality disorder (Benecke et al., 2009; Ehrenthal et al., 2012). To the authors’ knowledge, however, there is only one study that focused on correlations between structural impairment according to the OPD, and burnout (Bugaj et al., 2016). Here, medical students with higher structural impairments were more prone to stress and burnout than those with lower structural impairments.

**Table 1:** Definition of the dimensions of structural impairment.

	Self	Object
Perception	Self-perception	Perception of the other
	The ability to reflect and differentiate one’s self-image, to differentiate one’s affects and to have a sense of one’s own identity.	The ability to distinguish one’s own thoughts, needs and impulses from those of others, to perceive others holistically and realistically.
Regulation	Self-regulation	Regulation of relationships
	The ability to control one’s own impulses, to tolerate affect und to regulate one’s self-worth.	The ability to protect relationships, to balance interests and to anticipate the reaction of others.
Emotional communication	Internal emotional communication	Internal emotional communication
	The ability to experience affect, to use fantasies and to emotionally experience the bodily self.	The ability to establish emotional contact, to communicate affect and to empathize.
Attachment	Attachment to internal objects	Attachment to external objects
	The ability to internalize positive representations of others, to use positive introjects of others and to form variable bonds.	The ability to attach to others, to accept help and to detach from relationships

Source: OPD Task Force (2011).

In addition to the personality structure, conflicts are defined as “unconscious inner-psychic clashes of opposing bundles of motives” (OPD Task Force, 2011), which have arisen in life history, persist over time and are a possible cause for the development of later psychological disorders (OPD Task Force, 2011). In the OPD, a total of seven conflicts are defined in addition to a category of repressed perception of conflict and emotions (K0): individuation vs. dependency conflict (K1), submission vs. control conflict (K2), need for care vs. self-sufficiency conflict (K3), self-worth conflict (K4), guilt conflict (K5), oedipal conflict (K6), identity conflict (K7). In addition, an active (a) and a passive (p) coping mode is described for each of the seven conflicts. These comprise conflict- and mode-specific self and object representations, which in turn are reflected in the experience and behavior in relation to important aspects of life (i.e., profession, social environment, the body etc.). Table 2 provides an overview of the definitions of the OPD conflicts as well as their corresponding conflict modes and the category K0 (OPD Task Force, 2011). The underlying psychodynamic concept of conflict incorporates the idea that the frustration of desires, needs, motives or ideas by existing values and norms is so unbearable that the corresponding contents must be warded off and kept away from consciousness (OPD Task Force, 2011). If these experiences remain unprocessed, they manifest themselves in an unconscious neurotic conflict (Schneider et al., 2008). In addition, the development of a neurotic conflict, whether in the active or passive mode, involves the fixation on a rigid or unresolvable either-or situation, which fails to result in resolution or decision (OPD Task Force, 2011). Since unconscious conflicts cannot be measured directly, the OPD attempts to capture them on the basis of their outward manifestations in experience and behavior. The unconscious conflicts are then assessed through the individual experiences and behaviors (OPD Task Force, 2011). For example, if a person’s need for dependency was frustrated in early childhood, that person may develop a lifelong fear of dependency. This fear must be kept away from consciousness through corresponding experiences and behaviors. As a result, the person may excessively strive for individuation (K1a) to avoid feelings of dependency of other people. The frustration of one’s need for dependency is thus reflected in its flip side, namely an unbalanced striving for individuation (K1a) (OPD Task Force, 2011). Even though these inner-psychic, unconscious conflicts and their forms of coping aren’t necessarily pathogenic, they represent a vulnerability factor for mental disorders (OPD Task Force, 2011). In general, it was shown, that most of the conflict modes were positively associated with symptom load (Benecke et al., 2018) and job related burnout problems (Gisch et al., 2020). In contrast, the self-rated conflict modes of an exaggerated sense of self-worth (K4a) and of dismissing guilt (K5a) were negatively correlated with subjective symptom load (Benecke et al., 2018) and burnout problems (Gisch et al., 2020). The category repressed perception of conflict and emotions (K0) was not related to symptom load (Benecke et al., 2018) but was negatively correlated with job related problems (Gisch et al., 2020).

**Table 2:** Definition of the OPD category repressed perception of conflict and emotions and the conflict modes.

OPD-Conflict	Definition
K0 repressed perception of conflict and emotions	This category is used to assess people who have difficulty perceiving conflicts and feelings in themselves and in their relationships with others.
K1 individuation vs. dependency conflict	In this conflict the main fear concerns having close relationships. While the active mode is characterized by the strive for individuation and distance (K1a) the passive mode comprises the longing for close relationships (K1p).
K2 submission vs. control conflict	This conflict concerns insecurities in relation to the feeling of agency and control. While the active mode is characterized by controlling and dominating others (K2a), the passive mode, however, is characterized by submissive, often passive-aggressive behavior (K2p).
K3 need for care vs. self-sufficiency conflict	This conflict comprises insecurities about the amount of need for care in relationships and their defense. The active mode is characterized by modesty and caring for others (K3a). The passive mode, on the other hand, is characterized by needy, clingy, and demanding behavior (K3p).
K4 self-worth conflict	In this conflict the worthiness of a person is at risk (K4). While the active mode is expressed by a strong sense of self and the devaluation of others (K4a), the passive mode is characterized by the devaluation of oneself (K4p).
K5 guilt conflict	This conflict is about uncertainties in accepting responsibility and guilt. In the active mode one dismisses any kind of guilt and blames others (K5a), in the passive mode, however, one takes the blame (K5p).
K6 oedipal conflict	This conflict oscillates around insecurities regarding (gender-) role-identities, which either results the need for attention, admiration and recognition as a man or woman (K6a) or the avoidance and suppression of it (K6p).
K7 identity conflict	This conflict refers to one’s own sense of identity. Whereas the active mode is characterized by overacting of one’s own lack of identity (K7a), in the passive mode a feeling of lack of identity predominates (K7p)

Source: OPD Task Force (2011).

Conflicts have always been at the center of classical psychoanalytic theorizing (Freud, 1952 (1895)). According to the OPD, however, the conflict modes are dimensionally related to structural impairment (OPD Task Force, 2011). While the conflict modes measure psychoanalytically substantial aspects of personality, structural impairment defines the capacity to process internal and external stressors (OPD Task Force, 2011). In this regard, the OPD Diagnosis system may bear parallels to the alternative model of personality disorder in the DSM-5 (Zimmermann et al., 2012, 2013, 2015a,b; American Psychiatric Association, 2013; Benecke and Henkel, 2021). In the DSM-5, each of the personality disorders is defined on the basis of maladaptive personality traits (criterion B) and the Level of Personality Functioning (LPF) (criterion A) (American Psychiatric Association, 2013). In the OPD, on the other hand, the way in which conflict modes are processed is determined by the level of structural impairments, thus ranging from subclinical conflict tensions, to neurotic, repetitive conflicts, to conflict schemas in the presence of severe structural impairments. Thus, the more severe the structural impairments, the more difficult it is to identify stable conflict patterns (OPD Task Force, 2011). In this regard, one study showed that as structural impairment increases, conflicts become harder to delineate (Henkel et al., 2022).

Up to date, there is only one study each that examined the relationship between structural impairment and burnout (Bugaj et al., 2016) and between the conflict modes and job related burnout problems (Gisch et al., 2020). The main object of the present study is to explore burnout and the influence of individual factors among the German working population.

Based on previous findings, it was expected that structural impairment is positively related to burnout (hypothesis I). Regarding the associations between the conflict modes and burnout, it was assumed that all conflict modes, except the conflict modes K4a and K5a, are positively related to burnout (hypothesis II). To the author’s knowledge, there is no study that examined the incremental validity of structural impairment beyond the conflict modes in relation to burnout. Therefore, we aimed to provide new insight into the incremental validity of structural impairment on burnout. In this regard, we expected that structural impairment would predict burnout beyond the conflict modes and the category K0 (hypothesis III). Moreover, no study has yet examined the mediating effects of structural impairment on the relationships between the conflict modes and burnout. Although the conflict modes are generally positively related to burnout, structural impairment in particular has an impact on how individuals regulate themselves and their relationships to others. Therefore, we assumed that structural impairment reduces the influence of the conflict modes on burnout. Based on the theoretical considerations and the empirical findings presented, we hypothesized that structural impairment mediates the associations between the conflict modes and burnout (hypothesis IV).

## 2. Materials and methods

### 2.1. Study design

The study was approved by the ethics committee of the Charité Universitätsmedizin Berlin (ID: EA4/180/21). The present cross-sectional study was conducted on a sample collected as part of a validation study in August 2018 (Gisch et al., 2020). A non-probabilistic quota sample was used to obtain a broad cross-section of the German working population regarding gender, age, and place of residence. The corresponding quotas were calculated using the population update for 2016 based on the 2011 census of the Federal Statistic Office (2018). In terms of statistical power, we followed the recommendations of (Stevens, 2009) for multiple regression analyses. According to Stevens (2009), a minimum number of 15 subjects per predictor variable is required. Following this rule and 17 predictor variables, a minimum of 255 observations was estimated for the present study. In addition, we followed the sample sizes estimates by Fritz and MacKinnon (2007) for bias-corrected mediation analyses. Our sample size estimates were based on the small to medium effects of previous mediation analyses examining constructs similar to those examined in the present study (Bugaj et al., 2016; Di Fabio and Saklofske, 2021). Accordingly, a sample size of 400 subjects is recommended. Since about 25% incomplete records were anticipated, a case number of 500 was considered as a minimum. With a total sample size of 545 subjects in the present study, the sample requirements were exceeded.

Study participants were recruited *via* the crowdsourcing platform clickworker.com, with an expense allowance of €5.50 paid. All study participants provided fully informed written consent. Inclusion criteria for the evaluation were defined as follows: The study participants had to be between 20 and 65 years of age, had to agree to the conditions of participation and had to complete the questionnaire up to the last page. In addition, a Relative Speed Index (RSI) was defined (Leiner, 2013). If the RSI was below 2.0, the data were excluded from further data a*n*alysis due to an excessively fast completion of the questionnaire.

### 2.2. Sample

Table 3 presents an overview of the sociodemographic characteristics of the study participants. After excluding a total of 44 individuals (RSI < 2.0), the sample comprised a total of 545 individuals. The sample approximately reflected the German working population in terms of gender, age, and place of residence. Supplementary Table S1 shows the achieved quotas in the present sample in terms of gender, age and place of residence compared with the quotas calculated using the 2016 population update based on the 2011 census of the Federal Statistic Office (2018). In the present sample the gender distribution was approximately equal (48% women, 51% men). The age range was 20–65 years (*M* = 43.1, *SD* = 11.24). In terms of marital status, most study participants reported being either married or in a civil partnership (37.01%). Regarding the level of education, most of the study participants stated that they had completed grammar school (53.2%). In addition, a large proportion of the subjects stated, that they either completed an apprenticeship, vocational training, or technical school (45.86%). Finally, most of the study participant worked full- time (68.53%).

**Table 3:** Sociodemographic characteristics of participants.

	N	%
Total sample size	545	100
Gender		
Female	262	48.4
Male	279	51.6
Age		
20 years to under 35 years	136	25.9
25 years to under 50 years	200	38.1
50 years to under 65 years	189	36
Level of education		
High School	287	53.2
Technical College	99	18.3
Extended High School	3	0.6
Polytechnical High School	19	3.5
Secondary School	98	18.2
Basic School	34	6.3
No school	0	0
Employment		
Full-time (> 30 h per week)	355	68.53
Part-time (15–30 h per week)	116	22.39
Marginally employed (< 15 h per week)	47	9.07

*N* = Sample size.

### 2.3. Materials

#### 2.3.1. The short version of the OPD structure questionnaire

The OPD-SQS (Ehrenthal et al., 2015) is a screening instrument for the assessment of structural impairments. It was developed from the original 95-item long version, the OPD-SQ (Ehrenthal et al., 2012), using a statistical-exploratory procedure (Ehrenthal et al., 2015). The OPD-SQS (Ehrenthal et al., 2015) includes a total of 12 items and 3 subscales. The subscales self-perception, relationship model, and contact behavior are assessed with 4 items each.

The self-perception subscale includes items from the domains identity, self-reflection, affect differentiation, and affect tolerance of the OPD-SQ. Thus, the self-perception subscale integrates aspects of the self with emotion regulation skills. The relationship model subscale includes items from the domains internalizing, self-object differentiation, and realistic object perception of the OPD-SQ. Accordingly, the relationship model subscale links representations of relationship experiences to corresponding expectations of new relationships. Finally, the contact behavior subscale includes items from the domains affect communication, contact establishment, anticipation, and self-esteem regulation of the OPD-SQ, thus mapping both interactional skills and aspects of self-uncertainty (Ehrenthal et al., 2015). Items are rated on a five-point Likert scale ranging from “strongly disagree” (0) to “strongly agree” (4). Because the OPD-SQS was used for the first time on a sample covering a broad cross-section of the German working population, a confirmatory factor analysis (CFA) was calculated (Supplementary Figure S1). For the determination of the internal consistency McDonald’s ω was used (McDonald, 1999). McDonald’s ω was interpreted as satisfactory (ω ≥ 0.7) or good (ω ≥ 0.8) (Bland and Altman, 1997). Overall, the fit indices of the three-factor model showed acceptable to good fit indices (CFI = 0.99; TLI = 0.99; RMSEA [90% CI] = 0.13 [0.12, 0.14]; SRMR = 0.08). Furthermore, the internal consistency of the OPD-SQS total score was ω = 0.90 in the present study and can be considered good. The reliability values of the subscales of the OPD-SQS can be considered satisfactory to good with ω values between 0.78 and 0.85.

#### 2.3.2. The OPD conflict questionnaire

The conflicts (K1–K6) and their coping modes (active, passive) as well as the category K0 were measured with the OPD-CQ (Benecke et al., 2018). This self-report questionnaire includes a total of 14 scales and 66 items. In addition to the category repressed perception of conflict and emotions (K0), the OPD-CQ defines the individuation versus dependency conflict (K1), the submission versus control conflict (K2), the need for care versus self-sufficiency conflict (K3), the self-worth conflict (K4), the guilt conflict (K5), and the oedipal conflict (K6). During the development of the OPD-CQ, the items capturing the identity conflict (K7) did not meet the required scoring criteria, so it was not included in the questionnaire (Benecke et al., 2018). In addition, the OPD-CQ defines the corresponding active and passive coping mode for each conflict. Items are rated using a five-point Likertscale ranging from “strongly disagree” (0) to “strongly agree “(4). For the scales K1a, K3p, K4a, K4p, K5p, K6a, and K6p, satisfactory to good values in terms of internal consistency were found in a preliminary study (ω = 0.74–0.86) (Gisch et al., 2020). The scales K0, K1p, K2a, K2p, K3a, and K5a did not show satisfactory internal consistency (ω < 0.70). For the scales K2a and K2p, which had unacceptable values in terms of internal consistency (ω < 0.50), the additional items provided by Benecke et al. (2018) were used. As a result, the internal consistencies of these scales improved somewhat, but were still in the unsatisfactory range (ω = 0.52–0.68). Furthermore, a confirmatory factor analysis (CFA) was conducted to check the dimensionality and the fit indices CFI and RMSEA for the individual scales. All scales, except the scales K2a and K0, achieved at least acceptable to good fit indices (Gisch et al., 2020).

#### 2.3.3. The burnout screening scales I and II

The two Burnout Screening Scales (BOSS-I/-II) were used for self-assessment of symptoms occurring in the context of a burnout syndrome (Geuenich and Hagemann, 2014). The BOSS-I measures the subscales job, self, family, and friends with a total of 30 Items. The job subscale measures job-related burnout problems on an emotional, cognitive, and behavioral level, some of which are directly related to external working conditions. The self-subscale, on the other hand, captures the self, the individuality, and the overall individual situation on an emotional, cognitive, or physical level. Both scales comprise 10 items each. While the family subscale measures conflicts, tensions, alienation or neglect of relationships within the family or partnership, the friends subscale measures social withdrawal, problems in drawing boundaries or disinterest in friends. The family subscale and the friends subscale contain 5 items each. The BOSS-II, on the other hand measures a person’s physical, cognitive, and emotional problems with 10 items each. While the physical problem subscale assesses illnesses and physical impairments, focusing on the cardiovascular system, the cognitive problems subscale assesses difficulties in cognitive performance as well as attitudes and evaluations of oneself. Finally, the emotional problems subscale primarily measures stress-related emotional states. While the BOSS-I primarily focusses on systemic aspects of burnout, the focus of the BOSS-II is on the three observational levels of psychosomatic burnout problems (Geuenich and Hagemann, 2014). The items are rated on a five-point Likert scale ranging from “strongly disagree” (0) to “strongly agree” (4). Satisfactory psychometric criteria were reported for the BOSS-I and the BOSS-II (Geuenich and Hagemann, 2014). In the present study, the internal consistency of the subscales of the BOSS-I (ω = 0.87–0.95) and the BOSS-II (ω = 0.90–0.96) were good. Besides, the internal consistency of the total scales of the BOSS-I and the BOSS-II, each of which represents the average burnout symptomatology, was also good (ω = 0.97).

### 2.4. Data analysis

Statistical analysis was performed using SPSS 20 and R (Version 3.5.1 – “Feather Spray,” R Core Team, 2018). First, descriptive data were computed and presented as means, standard deviations and Pearson’s correlation coefficients. Bonferroni-corrected significance levels were used for multiple testing (*p* < 0.05/50 = 0.001) (Shaffer, 1995). According to Cohen (1988), the correlation coefficients were interpreted as marginal (*r* < 0.1) small (*r* ≥ 0.1), medium (*r* ≥ 0.3) and large (*r* ≥ 0.5). The prerequisites for the regression analyses were analyzed. Multicollinearity was analyzed by means of tolerance (> 0.10) and the variance inflation value (< 10.0) after which multicollinearity could be ruled out (Cohen et al., 2003). In addition, the residuals were tested for homoscedasticity using the Breusch-Pagan test (Breusch and Pagan, 1979). To deal with single violations of homoscedasticity, boot-strapping methods were used in the following statistical analyses.

Second, to examine whether structural impairment improves the explanatory contribution of the conflict modes on burnout, after controlling for gender, age, and level of education, hierarchical regression analyses were conducted for the BOSS-I and the BOSS-II. To obtain robust results, bootstrapping and bias corrected and accelerated confidence intervals with 10,000 iterations and 95% confidence intervals were used. In this process, the predictors were successively included in the hierarchical regression models. To control for the sociodemographic variables gender, age, and level of education were added in a first step. In addition, the 13 facets of the OPD-CQ were added in a second step and the OPD-SQS total score was included in a third step. To test whether structural impairment adds value in addition to the conflict modes in BOSS-I and BOSS-II after controlling for gender, age, and level of education, these two models were also compared using an F-test. In addition, Cohen’s *f*^2^ was calculated based on the adjusted determinant to interpret the strength of the incremental effects (Cohen, 1988). The effects were interpreted as marginal (*f*^2^ < 0.02), small (*f*^2^ ≥ 0.02), medium (*f*^2^ ≥ 0.15) or large (*f*^2^ ≥ 0.35) (Cohen, 1988).

**Figure 1:**
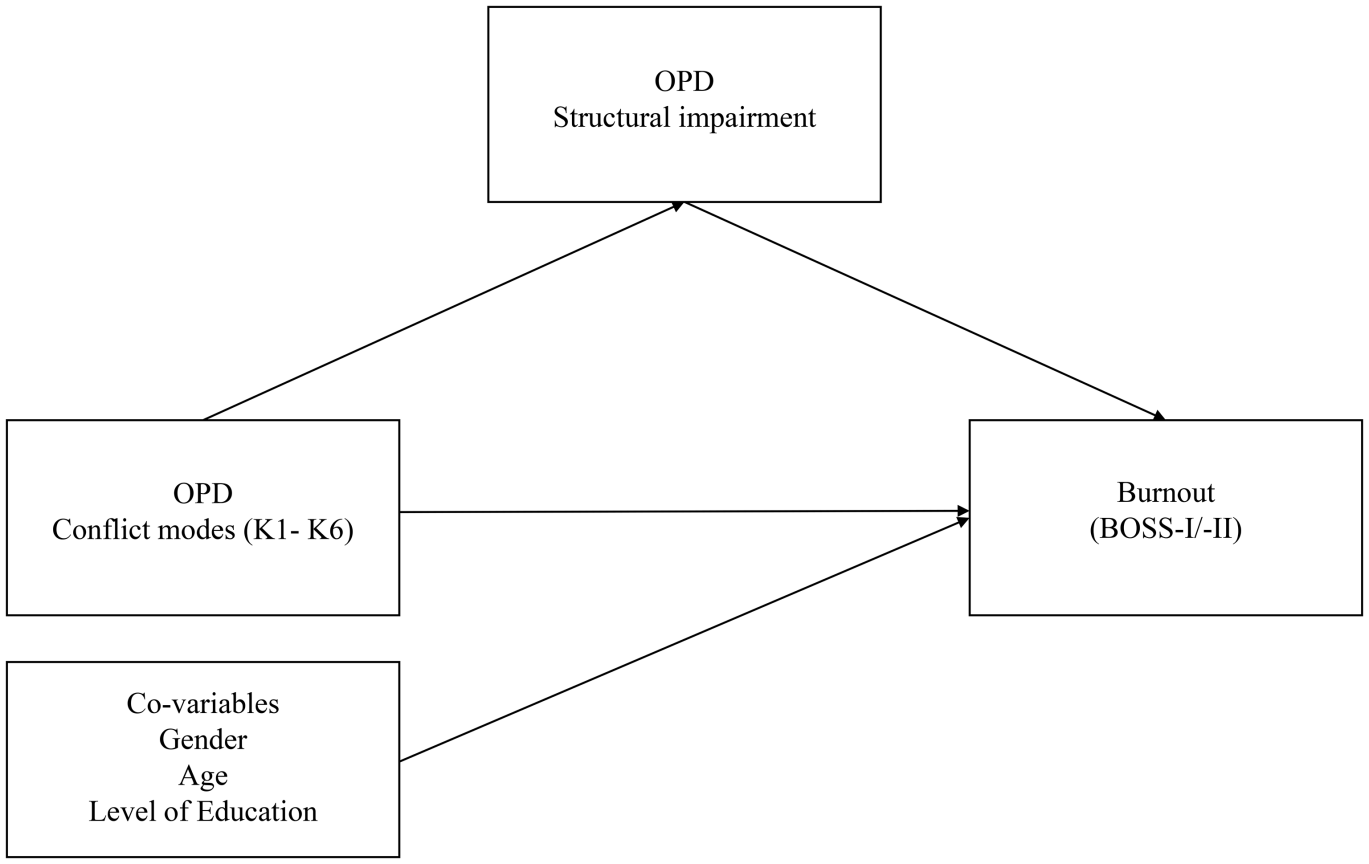
Theoretical model for the relationships between structural impairment, conflict modes and burnout.

Third, mediation analyses were performed to explore whether structural impairment mediated the impact of the conflict modes (K1a/p – K6a/p) and the category K0 on each of the two burnout screening scales (BOSS-I/-II) (see Figure 1). For the OPD-CQ all 12 conflict modes and the category K0 were included in the mediation analysis. Since the mediation analysis provides for three regression analyses for each constellation, structural impairment was operationalized by the OPD-SQS total score. For each, the BOSS-I and the BOSS-II, the total scores were used. However, a conflict-specific mediation analysis was only computed when the correlative effects between structural impairment, the conflict mode and burnout were at least small (r ≥ 0.1). Furthermore, gender, age, and level of education were included as covariates in the mediation analysis to exclude possible confoundation by sociodemographic data. Mediation analyses were performed using PROCESS macros v3.4 for SPSS (Hayes, 2018), which uses linear least squares regression to calculate unstandardized path coefficients of the total, direct, and indirect effects. The bootstrapping method of Preacher and Hayes (2004) with 5,000 iterations was used to calculate the inferential statistics and 95% confidence intervals. Indirect effects were considered significant if the confidence interval did not include zero.

## 3. Results

### 3.1. Descriptive statistics

Due to the lack of one-dimensionality of the scales K2a and K0, no interpretations of the related results were made (Gisch et al., 2020). First the intercorrelations are presented. Positive correlations were found between structural impairment and burnout (BOSS-I: *r* = 0.62, *p* < 0.01; BOSS-II: *r* = 0.65, *p* < 0.01). Besides, positive correlations were found between the conflict modes K1a, K1p, K2p, K3a, K4p, K5p, K6a, and K6p (BOSS-I: *r* = 0.14–0.66, *p* < 0.01; BOSS-II: *r* = 0.11–0.68, *p* < 0.05, respectively, p < 0.01) and burnout. Moreover, negative correlations were found between the conflict modes K4a and K5a and burnout (BOSS-I: *r* = − 0.21, *p* < 0.01; BOSS-II: r = −0.32, −0.30, *p* < 0.01). However, no correlations were found between the conflict mode K3p and burnout (BOSS-I: *r* = 0.00, *p* > 0.05; BOSS-II: *r* = 0.03, *p* > 0.05). Furthermore, positive correlations were found between structural impairment and the conflict modes K1a, K1p, K2p, K3a, K4p, K5p and K6p (*r* = 0.22–0.75, *p* < 0.01). In contrast, negative correlations between structural impairment and the conflict modes K4a and K5a (*r* = −0.22, −0.30, p < 0.01) were found. In addition, structural impairment showed no correlation with the conflict mode K3p (*r* = 0.04, *p* > 0.05). Finally, structural impairment was only marginally correlated with the conflict mode K6a (*r* = 0.09, *p* < 0.05). Since the correlations between structural impairment and the conflict modes K3p and K6a were either not existent (K3p) or significant but marginal (K6a), conflict-specific mediation analyses were not computed for these two conflicts modes. The Pearson intercorrelation with Bonferroni-corrected significance levels as well as the mean scores and the SDs for each of the variables are presented in Table 4.

**Table 4:** Descriptive statistics and intercorrelations (*N* = 545).

Variable	*M*	SD	1	2	3	4	5	6	7	8	9	10	11	12	13	14	15	16
1. K0	1.94	0.53	1															
2. K1a	1.81	0.83	0.18^***^	1	–	–	–	–	–	–	–	–	–	–	–	–	–	–
3. K1p	1.45	0.69	−0.05	0.07	1	–	–	–	–	–	–	–	–	–	–	–	–	–
4. K2a	2.11	0.52	0.18^***^	0.09^*^	0.07	1	–	–	–	–	–	–	–	–	–	–	–	–
5. K2p	1.88	0.62	0.16^***^	0.33^***^	0.45^***^	0.08	1	–	–	–	–	–	–	–	–	–	–	–
6. K3a	2.37	0.58	0.14^**^	0.17^***^	0.18^***^	0.07	0.32^***^	1	–	–	–	–	–	–	–	–	–	–
7. K3p	2.12	0.71	0.01	−0.26^***^	0.27^***^	0.09^*^	0.10^*^	−0.01	1	–	–	–	–	–	–	–	–	–
8. K4a	1.68	0.69	0.08	−0.10^*^	−0.05	0.52^***^	−0.19^***^	−0.13^**^	0.01	1	–	–	–	–	–	–	–	–
9. K4p	1.09	0.91	−0.03	0.39^***^	0.50^***^	−0.11^*^	0.53^***^	0.26^***^	0.05	−0.36^***^	1	–	–	–	–	–	–	–
10. K5a	1.64	0.55	0.16^***^	0.08	−0.16^***^	0.28^***^	−0.08	−0.19^***^	−0.04	0.36^***^	−0.30^***^	1	–	–	–	–	–	–
11. K5p	1.47	0.77	−0.03	0.21^***^	0.48^***^	0.05	0.41^***^	0.37^***^	0.11^**^	−0.10^*^	0.59^***^	−0.40^***^	1	–	–	–	–	–
12. K6a	1.4	0.63	−0.03	0.01	0.36^***^	0.37^***^	0.09^*^	−0.06	0.19^***^	0.43^***^	0.06	0.13^**^	0.17^***^	1	–	–	–	–
13. K6p	1.88	0.73	0.17^***^	0.34^***^	0.15^***^	−0.19^***^	0.45^***^	0.34^***^	−0.12^***^	−0.44^***^	0.43^***^	−0.18^***^	0.33^***^	−0.45^***^	1	–	–	–
14. OPD-SQS	1.76	0.77	−0.02	0.48^***^	0.46^***^	0	0.58^***^	0.29^***^	0.04	−0.30^***^	0.75^***^	−0.22^***^	0.56^***^	0.09^*^	0.44^***^	1	–	–
15. BOSS-I	1.38	0.92	−0.03	0.39^***^	0.44^***^	−0.03	0.47^***^	0.19^***^	0	−0.21^***^	0.66^***^	−0.21^***^	0.48^***^	0.14^**^	0.29^***^	0.62^***^	1	–
16. BOSS-II	1.24	1.01	0.08	0.38^***^	0.42^***^	−0.06	0.43^***^	0.22^***^	0.03	−0.30^***^	0.68^***^	−0.23^***^	0.44^***^	0.11^*^	0.29^***^	0.65^***^	0.90^***^	1

M, mean; SD, standard deviation; the values shaded in gray do not represent satisfactory model performance regarding the verification of the one-dimensionality of the scale; K0, repressed perception of conflict and emotions; K1a, individuation vs. dependency conflict in active mode; K1p, individuation vs. dependency conflict in passive mode; K2a, submission vs. control conflict in active mode; K2p, submission vs. control conflict in passive mode; K3a, need for care vs. self-sufficiency conflict in active mode; K3p, need for care vs. self-sufficiency conflict in passive mode; K4a, self-worth conflict in active mode; K4p, self-worth conflict in passive mode; K5a, guilt conflict in active mode; K5p, guilt conflict in passive mode; K6a, oedipal conflict in active mode; K6p, oedipal conflict in passive mode; SQS, short version of the OPD – Structure Questionnaire total score; BOSS-I, burnout total score (job, self, family, and friends); BOSS-II, burnout total score (physical, cognitive, and emotional).

*p < 0.05, **p < 0.01 (Bonferroni corrected *p* value), ***p < 0.001.

### 3.2. Incremental validity

To investigate the incremental contribution of structural impairment beyond the conflict modes, after controlling for gender, age, and level of education, hierarchical regression analyses were computed. It was shown that the regression models including sociodemographic data, conflict modes and structural impairment showed a significantly better prediction of burnout (BOSS-I/-II) in comparison with the models without the OPD-SQS total score (BOSS-I: *f*^2^ = 0.01, *b* = 0.18, 95% BCa CI [0.05, 0.33], *p* < 0.01; BOSS-II: *f*^2^ = 0.04, *b* = 0.30, 95% BCa CI [0.16, 0.45], *p* < 0.001). The proportion of variance increased with respect to burnout by 0.7 to 51.0% in BOSS-I and by 1.7 to 54.6% in the BOSS-II when the OPD-SQS total score was added to the models. In Tables 5, 6 an overview of the results of the hierarchical regression analyses can be found for the BOSS I (Table 5) and the BOSS-II (Table 6).

**Table 5:** Hierarchical regression results for burnout (BOSS-I).

Variable	B	95% BCa CI^a^		SE^a^	*β*	*R* ^2^	∆*R*^2^
		LL	UL				
Step 1						0.023	0.023**
Constant	2.13***	1.58	2.48	0.25			
Gender	−0.01**	−0.02	0	0	−013**		
Age	0	−0.1	0.24	0.08	0		
Level of Education	−0.06	−0.1	−0.01	0.02	−0.11		
Step 2						0.5	0.48***
Constant	0.74*	0.44	1.27	0.33			
Gender	0	−0.01	0.01	0	−0.01		
Age	−0.01	−0.1	0.24	0.09	−0.01		
Level of Education	−0.04*	−0.07	0	0.02	−0.08*		
K0	−0.05	−0.19	0.04	0.06	−0.03		
K1a	0.20***	0.1	0.3	0.05	0.18***		
K1p	0.14*	0	0.25	0.07	0.1		
K2a	−0.03	−0.17	0.1	0.07	−0.02		
K2p	0.17**	0.05	0.33	0.07	0.11**		
K3a	−0.07	−0.18	0.06	0.06	−0.04		
K3p	−0.05	−0.14	0.06	0.05	−0.04		
K4a	−0.02	−0.15	0.11	0.07	−0.01		
K4p	0.44***	0.34	0.54	0.05	0.43***		
K5a	−0.08	−0.21	0.04	0.07	−0.05		
K5p	0.12*	0	0.23	0.06	0.10*		
K6a	0.05	−0.08	0.18	0.07	0.04		
K6p	−0.07	−0.2	0.05	0.06	−0.05		
Step 3						0.51	0.01***
Constant	0.58	−0.14	1.2	0.34			
Gender	0	−0.01	0.01	0	0.01		
Age	0	−0.08	0.23	0.09	0		
Level of Education	−0.03*	−0.07	0	0.02	−0.07*		
K0	−0.03	−0.17	0.08	0.06	0.02		
K1a	0.16**	0.05	0.26	0.05	0.14**		
K1p	0.13*	0	0.25	0.07	0.09*		
K2a	−0.05	−0.17	0.09	0.07	−0.03		
K2p	0.13*	−0.01	0.28	0.07	0.09*		
K3a	−0.07	−0.18	0.05	0.06	−0.04		
K3p	−0.05	−0.15	0.05	0.05	−0.04		
K4a	0	−12	0.13	0.06	0		
K4p	0.38***	0.26	0.49	0.06	0.38***		
K5a	−0.07	−0.21	0.06	0.07	−0.04		
K5p	0.1	−0.02	0.21	0.06	0.08		
K6a	0.04	−0.1	0.18	0.07	0.03		
K6p	−0.08	−0.21	0.05	0.07	−0.06		
SQS	0.18**	0.05	0.32	0.07	0.15**		

a Confidence intervals and standard errors per BCa-Bootstrapping with 10,000 iterations; the values shaded in gray do not represent satisfactory model performance regarding the verification of the one-dimensionality of the scale; BOSS-I, burnout total score (job, self, family, and friends); BOSS-II, burnout total score (physical, cognitive, and emotional); K0, repressed perception of conflict and emotions; K1a, dependence vs. individuation conflict in active mode; K1p, dependence vs. individuation conflict in passive mode; K2a, submission vs. control conflict in active mode; K2p, submission vs. control conflict in passive mode; K3a, need for care vs. self-sufficiency conflict in active mode; K3p, need for care vs. self-sufficiency conflict in passive mode; K4a, self-worth conflict in active mode; K4p, self-worth conflict in passive mode; K5a, guilt conflict in active mode; K5p, guilt conflict in passive mode; K6a, oedipal conflict in active mode; K6p, oedipal conflict in passive mode; OPD-SQS, short version of the OPD – Structure Questionnaire total score.

Adjusted *R*^2^ = 0.18 (*p* < 0.01) for step 1; adjusted *R*^2^ = 0.49 for step 2 (*p* < 0.001); adjusted *R*^2^ = 0.49 (*p* < 0.01) for step 3; *p < 0.05, **p < 0.01, and ***p < 0.001.

**Table 6:** Hierarchical regression results for burnout (BOSS-II).

Variable	B	95% BCa CI^a^		SE^a^	β	R	∆R2
		LL	UL				
Step 1						0.023	0.023**
Constant	2.03***	1.48	2.45	0.26			
Gender	−0.01	−0.01	0	0	−0.07		
Age	−0.05	−0.14	0.15	0.08	−0.03		
Level of Education	−0.08**	−0.13	−0.03	0.03	−0.14		
Step 2						0.53	0.51***
Constant	0.63*	−0.12	1.28	0.35			
Gender	0	0	0.01	0	0.05		
Age	−0.02	−0.11	0.2	0.09	−0.02		
Level of Education	−0.06**	−0.1	−0.02	0.02	−0.1		
K0	−0.13*	−0.27	−0.01	0.07	−0.07		
K1a	0.21***	0.11	0.31	0.05	0.17		
K1p	0.12	−0.03	0.24	0.07	0.08		
K2a	0.02	−0.13	0.18	0.08	0.01		
K2p	0.1	−0.04	0.24	0.07	0.06		
K3a	0.01	−0.11	0.14	0.06	0		
K3p	0.01	−0.1	0.11	0.05	0		
K4a	−0.15*	−0.3	0	0.08	−0.1		
K4p	0.57***	0.46	0.69	0.06	0.52		
K5a	−0.08	−0.23	0.07	0.07	−0.04		
K5p	0.03	−0.09	0.16	0.07	0.03		
K6a	0.07	−0.08	0.2	0.07	0.04		
K6p	−0.09	−0.22	0.03	0.07	−0.07		
Step 3						0.55	0.02***
Constant	0.36	−0.43	1.02	0.37			
Gender	0.01*	0	0.01	0	0.07		
Age	−0.01	−0.1	0.21	0.09	−0.01		
Level of Education	−0.05**	−0.09	−0.01	0.02	−0.09		
K0	−0.09	−0.23	0.02	0.07	−0.05		
K1a	0.14*	0.03	0.25	0.06	0.12		
K1p	0.1	−0.05	0.23	0.07	0.07		
K2a	0	−0.15	0.15	0.08	0		
K2p	0.03	−0.11	0.18	0.07	0.02		
K3a	0.01	−0.11	0.14	0.06	0		
K3p	0	−0.09	0.1	0.05	0		
K4a	−0.11	−0.25	0.03	0.07	−0.08		
K4p	0.48***	0.36	0.6	0.06	0.43		
K5a	−0.07	−0.21	0.07	0.07	−0.04		
K5p	0	−0.12	0.13	0.06	0		
K6a	0.05	−0.1	0.18	0.07	0.03		
K6p	−0.12	−0.25	0.01	0.07	−0.08		
SQS	0.30***	0.16	0.45	0.07	0.23		

a Confidence intervals and standard errors per BCa-Bootstrapping with 10,000 iterations; the values shaded in gray do not represent satisfactory model performance regarding the verification of the one-dimensionality of the scale; BOSS-I, burnout total score (job, self, family, and friends); BOSS-II, burnout total score (physical, cognitive, and emotional); K0, repressed perception of conflict and emotions; K1a, dependence vs. individuation conflict in active mode; K1p, dependence vs. individuation conflict in passive mode; K2a, submission vs. control conflict in active mode; K2p, submission vs. control conflict in passive mode; K3a, need for care vs. self-sufficiency conflict in active mode; K3p, need for care vs. self-sufficiency conflict in passive mode; K4a, self-worth conflict in active mode; K4p, self-worth conflict in passive mode; K5a, guilt conflict in active mode; K5p = guilt conflict in passive mode; K6a = oedipal conflict in active mode; K6p = oedipal conflict in passive mode; OPD-SQS = short version of the OPD – Structure Questionnaire total score.

Adjusted *R*^2^ = 0.02 for step 1 (*p* < 0.01); adjusted *R*^2^ = 0.51 (*p* < 0.001) for step 2; adjusted *R*^2^ = 0.53 (*p* < 0.001 for step 3); *p < 0.05, **p < 0.01, and ***p < 0.001.

### 3.3. Mediation analysis

It was shown that the conflict modes K1a, K1p, K2p, K3a, K4p, K5p, and K6p were positively associated with burnout, and the conflict modes K4a and K5a were negatively associated with burnout (s.a). Depending on the conflict-specific mediation models, these associations decreased or disappeared fully when structural impairment was introduced as a mediator (Table 7). The direct associations between the conflict modes and burnout disappeared fully only in the mediation models of the conflict modes K1a, K2p, K3a, K4a, and K6p. The associations between the conflict modes K3a and K6p were fully mediated by structural impairment for both the BOSS-I and the BOSS-II. However, the models of the conflict modes K1a and K2p were fully mediated only with respect to the BOSS-II, and the model of the conflict mode K4a was fully mediated only with respect to the BOSS-I. Partial mediations with respect to both the BOSS-I and the BOSS-II, were found for the models of the conflict modes K1p, K4p, K5a, and K5p. However, the models of the conflict modes K1a and K2a were partially mediated only with respect to the BOSS-I, and the model of K4a was partially mediated only with respect to the BOSS-II. For almost all partial mediation models, the direct effect was reduced by at least half after structural impairment was included in the models. The only exception is the model of the conflict mode K4p. Here, the direct effect proved to be stronger than the indirect effect. A tabular overview of all results can be found in Supplementary Table S2.

**Table 7:** Total, direct and indirect effects.

			BOSS-I							BOSS-II				
Variable	Path	*B*	*B* SE	*β*	*t*	*p*	95% CI	Path	*B*	*B* SE	*β*	*t*	*p*	95% CI
K1a	c’	0.14	0.05	0.12	2.65	0.01	[0.03, 0.24]	c’	0.1	0.05	0.08	1.88	0.06	[0.00, 0.21]
	ab	0.31	0.03				[0.25, 0.38]	ab	0.37	0.04				[0.30, 0.45]
K1p	c’	0.25	0.06	0.19	4.1	<0.001	[0.13, 0.37]	c’	0.22	0.07	0.15	3.32	<0.001	[0.10, 0.35]
	ab	0.3	0.03				[0.24, 0.38]	ab	0.37	0.04				[0.29, 0.46]
K2p	c’	0.22	0.07	0.15	3.11	0	[0.08, 0.36]	c’	0.13	0.07	0.07	1.72	0.09	[−0.02, 0.27]
	ab	0.46	0.05				[0.37, 0.55]	ab	0.57	0.05				[0.48, 0.68]
K3a	c’	0.01	0.06	0.01	0.2	0.84	[−0.11, 0.13]	c’	0.04	0.07	0.02	0.55	0.58	[−0.10, 0.17]
	ab	0.26	0.05				[0.17,0.35]	ab	0.3	0.05				[0.20, 0.41]
K4a	c’	−0.04	0.06	−0.03	−0.63	0.53	[−0.15, 0.08]	c’	−0.15	0.06	−0.1	−2.54	0.01	[−0.27, −0.03]
	ab	−0.24	0.04				[−0.32, −0.17]	ab	−0.27	0.05				[−0.37, −0.19]
K4p	c’	0.47	0.05	0.46	8.81	<0.001	[0.37, 0.58]	c’	0.54	0.06	0.32	9.36	<0.001	[0.42, 0.64]
	ab	0.2	0.04				[0.12, 0.28]	ab	0.24	0.04				[0.16, 0.32]
K5a	c’	−0.14	0.07	−0.08	−2.15	0.03	[−0.27, −0.01]	c’	−0.18	0.07	0.1	−2.65	0.01	[−0.31, −0.05]
	ab	−0.22	0.04				[−0.31, −0.14,]	ab	−0.26	0.05				[−0.36, −0.16]
K5p	c’	0.23	0.06	0.2	4.22	<0.001	[0.12, 0.34]	c’	0.16	0.06	0.12	2.63	0.01	[0.04, 0.28]
	ab	0.32	0.03				[0.26, 0.39]	ab	0.41	0.04				[0.33, 0.48]
K6p	c’	0.02	0.06	0.01	0.32	0.75	[−0.09, 0.13]	c’	−0.01	0.05	0.01	−0.13	0.9	[−0.11, 0.10]
	ab	0.33	0.04				[0.25, 0.41]	ab	0.39	0.05				[0.30, 0.48]

BOSS-I, burnout total score (job, self, family, and friends); BOSS-II, burnout total score (physical, cognitive, and emotional); Cl, confidence interval; K1a, individuation vs. dependency conflict in active mode; K1p, individuation vs. dependency conflict in passive mode; K2a, submission vs. control conflict in active mode; K2p, submission vs. control conflict in passive mode; K3a, need for care vs. self-sufficiency conflict in active mode; K3p, need for care vs. self-sufficiency conflict in passive mode; K4a, self-worth conflict in active mode, K4p, self-worth conflict in passive mode; K5a, guilt conflict in active mode; K5p, guilt conflict in passive mode; K6a, oedipal conflict in active mode, K6p, oedipal conflict in passive mode.

*p* < 0.05, *p* < 01, corrected value of *p* (Bonferroni) *p* < 0.001.

## 4. Discussion

The aim of the present study was to investigate the relationships between structural impairment, the conflict modes and burnout. As expected, structural impairment was positively related to burnout (hypothesis I). The assumption regarding the positive correlations between the conflict modes and burnout could also be confirmed (hypothesis II). An exception is the conflict mode need for care (K3p), which was not related to burnout. Furthermore, the conflict modes strong sense of self-worth (K4a) and dismissing guilt (K5a) were negatively associated with burnout. In line with our expectations, structural impairment was found to have additional explanatory power beyond the conflict modes in relation to burnout (hypothesis III). Finally, structural impairment mediated the associations between the conflict modes and burnout in a sample of the German working population (hypothesis IV). This hypothesis could be confirmed for 9 of the 12 conflict modes.

Regarding the relations between structural impairment and burnout (hypothesis I), it was found that structural impairment may be a risk factor for burnout. Thus, people with structural impairments may have greater difficulties in regulating themselves and their relationships to others in stressful situations, which makes them more prone to burnout. These results are in line with previous studies that found positive correlations between structural impairment and various mental problems in general (Mestel et al., 2004; Benecke et al., 2009, 2018; Ehrenthal et al., 2012; Obbarius et al., 2019; Baie et al., 2020; Bröcker et al., 2020) and burnout in particular (Bugaj et al., 2016). A concept that is close to structural impairment is emotional intelligence (Jauk and Ehrenthal, 2020). Previous studies found negative correlations between emotional intelligence (low structural impairment) and burnout (Mérida-López and Extremera, 2017; D’Amico et al., 2020). In addition, emotional intelligence was positively related to job satisfaction (Brunetto et al., 2012; Butakor et al., 2021), job engagement (D’Amico et al., 2020) and perceived social support (Ju et al., 2015; Fiorilli et al., 2019). These studies suggest that mitigating structural impairments could help to promote mental health in the workplace.

Furthermore, the associations between the conflict modes and burnout were examined (hypothesis II). It was found that people who strive for individuation (K1a), dependency (K1p), submission (K2p), modesty (K3a) as well as people with a low sense of self-worth (K4p), people who take the blame (K5p), people who seek for admiration as a man or woman (K6a) – or those who avoid this (K6p) – were burnout-prone. In contrast, people characterized by needy, affectionate, and demanding behavior (K3p) did not exhibit burnout. Moreover, people with a strong sense of self-worth (K4a) and people who dismiss guilt (K5a) were less prone to burnout.

Particularly noteworthy is the absence of burnout in people characterized by needy, clingy, and demanding behavior (K3p), as well as the negative associations between people with a strong sense of self-worth (K4a) or people who dismiss guilt (K5a) and burnout in the present study. However, the conclusion that these modes of processing inner conflicts are not associated with burnout should not be readily drawn. Regarding the associations between needy, clingy, and demanding people (K3p), one study found positive associations with symptom load (Benecke et al., 2018), but in this mixed clinical and non-clinical sample, the increased level of distress as well as the lack of personal resources may have contributed to the absence of burnout among people with needy, clingy, and demanding behavior (K3p) (Gisch et al., 2020). With respect to the self-worth conflict (K4a) and the guilt conflict (K5a) in the active mode, Benecke et al. (2018) noted that these conflict modes conceptually overlap with the narcissistic (K4a) and the antisocial personality disorder (K5). It can be assumed that the people of the present study who have a strong sense of self-worth (K4a) and dismiss guilt (K5a) do not experience mental distress as long as they can maintain their conflict mode as a defense mechanism (Benecke et al., 2018). For example, the defenses of an individual who stabilizes his or her self-worth (K4a) through professional hyper-achievement could become fragile as soon as professional goals are threatened. In this regard, one study showed that grandiose narcissists are particularly vulnerable when they fail at work (Besser and Priel, 2010). In contrast to our findings, in previous studies positive associations between narcissistic (Barnett and Flores, 2016; Von Känel et al., 2017) and antisocial personality traits (Al-Yaaribi and Kavussanu, 2017), and burnout among non-clinical samples were found. Therefore, whether a strong sense of self-worth (K4a) and dismissing guilt (K5a) are adaptive or maladaptive personality traits cannot be clearly answered without considering professional context. In addition, the negative direction of the associations between structural impairment and the conflict modes strong sense of self-worth (K4a) and dismissing guilt (K5a) may also be due to the fact that narcissistic as well as guilt-externalizing individuals may tend to represent themselves better on self-report questionnaires in terms of faking good (MacNeil and Holden, 2006).

In addition, people with a passive processing of their inner conflict were generally more prone to burnout than individuals with an active processing mode. Exceptions are needy, clingy, and demanding people (K3p) and people who strive for individuation (K1a). While the first (K3p) were not associated with burnout, the second (K1a) were more prone to burnout than people who strive for control (K2a), modesty (K3a) and people who seek for admiration as a man or woman (K6a). This finding could be due to the fact that passive forms of processing inner conflict are more likely to be associated with negative emotionality, including anxiety, shame, guilt, anger and envy (Benecke et al., 2018). In this regard, one study found that the passive conflict modes partially overlap with the big five personality dimension “neuroticism,” which captures negative emotionality (Gisch et al., 2021).

These study results supplemented previous findings that examined associations between the conflict modes and symptom load in general (Benecke et al., 2018) and burnout in particular (Gisch et al., 2020). However, Gisch et al. (2020) only examined the associations between the conflict modes and job-related burnout problems while the present study found associations between the conflict modes and a broader conceptualization of burnout, including a broader definition of systemic as well as psychosomatic burnout problems.

Our main question concerned the incremental validity of structural impairment beyond the conflict modes, as well as potential mediations of the associations between the conflict modes and burnout by structural impairment. In both studies, the sociodemographic data of gender, age, and level of education were controlled for as possible confounding variables. Regarding the incremental validity (hypothesis III), structural impairment explained burnout beyond the conflict modes, after controlling for the sociodemographic data gender, age, and level of education. Although effects were either marginal or small, the improvement in prediction was significant and points to the need to consider structural capacities of individuals in burnout prevention and treatment. This study is the first to examine the incremental validity of structural impairment related to burnout across and the conflict modes after controlling for sociodemographic data, complementing previous OPD-SQS validation studies (Ehrenthal et al., 2015; Obbarius et al., 2019).

Finally, mediation analyses of structural impairment on the associations between the conflict modes and burnout were conducted (hypothesis IV). Since, with burnout, the correlations of the conflict modes need for care (K3p) and the need for attention, admiration and recognition as a man or woman (K6a) were not existent or marginal, no mediation analyses were performed here. In all other mediation models, structural impairment mediated the associations between the conflict modes and burnout either fully or partially. The influence of the conflict modes on burnout was reduced to a substantial degree by structural impairment. An exception is the model of the conflict mode low self-worth (K4p). Here, the direct effect was stronger than the indirect effect. According to this, the devaluation of oneself (K4p) has a greater influence on burnout than structural impairment. One reason for this effect could be that both the scale K4p of the OPD-CQ and the OPD-SQS capture self-esteem related aspects. Based on the present study, structural impairment can generally be considered a decisive and persistent vulnerability factor for burnout. In addition, the present findings suggest that people with structural impairments tend to have a subjectively higher conflict load. Conflict load, however, does not necessarily have to be the primary problem; it could also be an expression–or coping mechanism–of structural impairments in an individual. Furthermore, it may be tentatively presumed that the conflict modes have an impact on the development of burnout. However, this influence eclipses as soon as structural impairments are present. The results presented on the relationships between structural impairment, the conflict modes, and burnout can be used as a substantive basis for appropriate, psychoanalytically-based intervention programs. In the clinical context, uncovering unconscious conflict in patients with structural impairments may not be sufficient to strengthen the individual’s ability to cope with internal as well as external experiences of stress. In this case, focusing on structure-based (Rudolf, 2018), mentalization-based (Bateman and Fonagy, 2019), or intersubjective (Stolorow et al., 1995) psychotherapeutic interventions might be required to counteract a prolonged course of treatment – or even a discontinuation of treatment. To prevent burnout at work, burnout prevention programs should also not only focus on the identification and reflection of one’s own motivational schemas but specifically target and mitigate structural impairments. Previous studies have shown that the development of self- and relationship-regulation skills in intervention programs in the work context has a positive impact on the stress level (Nooryan et al., 2011; Pérez Escoda et al., 2012). Therefore, focusing on mitigating structural impairments in training or coaching programs for workers and managers could contribute to the prevention of burnout.

There are several methodological limitations to the present study: First, it should be noted that the present study does not allow conclusions to be drawn about specific occupational groups at risk for burnout. In addition, previous studies have shown that mental health problems are more prevalent in samples recruited through crowd-sourcing platforms than in the general population (Walters et al., 2018; Zimmermann et al., 2020). This could also be the case in the present sample, limiting the generalizability of the results of the present study. In the OPD, structural impairment is rated independently of the conflict modes. In one study, however, conceptual overlaps were found between the self-assessment instruments, OPD-SQS and the OPD-CQ (Gisch et al., 2021). Furthermore, by using the OPD-CQ an instrument was used that is currently under revision (Benecke et al., 2018; Gisch et al., 2020). Another limitation is the use of self-assessment instruments, which carry the risk of response bias – especially the risk of socially desirable response behavior (van de Mortel, 2008). However, even honest respondent’s insight into problematic personality aspects may be limited. This could be particularly relevant for the self-worth conflict (K4a) and the guilt conflict (K5a) in the active mode. Therefore, third-party assessment instruments or OPD interviews should be included in future studies. In addition, the present study is a cross-sectional study that does not allow causal inferences to be made about the temporal sequence of the relationships examined (Maxwell and Cole, 2007; Maxwell et al., 2011). Therefore, the results should be interpreted with caution. Longitudinal studies are needed to identify structural impairments and the conflict modes as two significant risk factors for the development of burnout.

In conclusion, the present study extends our understanding of the interaction between psychoanalytically based personality concepts and burnout. More precisely, it provides an empirical basis for the incremental contribution of structural impairment beyond the conflict modes as well as for the mediating role of structural impairment between the conflict modes and burnout. The study results suggest that structural impairment explains burnout beyond the conflict modes. In addition, it may be cautiously presumed that the influence of the conflict modes in relation to burnout is fully or partially eclipsed when structural impairments are present. Therefore, not only the individual conflict modes but rather the structural capabilities may be considered in the treatment and prevention of burnout. To better understand the relationships between structural impairment, the conflict modes and burnout, future studies should not only use third-party assessment instruments or OPD interviews, but also conduct longitudinal studies.

## Data availability statement

The data generated for this study are available from the corresponding author on reasonable request. Requests to access these datasets should be directed to jp@dynamind.com.

## Ethics statement

The studies involving human participants were reviewed and approved by the Ethics Committee of the Charité Universitätsmedizin Berlin (ID: EA4/180/21). The patients/participants provided their written informed consent to participate in this study.

## Author contributions

HG collected the data and was responsible for the study design. JP and HG performed the statistical analysis. JP wrote the draft and the paper with reviewing by HG, JE, CM, and TK. All authors contributed to the article and approved the submitted version.

## Funding

This work was funded by the Mind-Institute SE., and the Publication fund Charité.

## Conflict of interest

The authors declare that the submitted work was conducted in the absence of any personal, professional, or financial relationships that could be construed as a potential conflict of interest.

## Publisher’s note

All claims expressed in this article are solely those of the authors and do not necessarily represent those of their affiliated organizations, or those of the publisher, the editors and the reviewers. Any product that may be evaluated in this article, or claim that may be made by its manufacturer, is not guaranteed or endorsed by the publisher.
