# Supplementary material for: Structural impairment and conflict load as vulnerability factors for burnout – A cross-sectional study from the German working population
Source: Front Psychol. 2023 Jan 24;13:1000572. doi: 10.3389/fpsyg.2022.1000572 (PMC9912933; doi:10.3389/fpsyg.2022.1000572)
Supplement: Supplementary file 1 [file Table_1.DOCX]

| **Table S1** |  |  |  |  |  |
| --- | --- | --- | --- | --- | --- |
| *Descriptive statistics of the sample regarding age, gender and residence.* | | | | |  |
| Sample (N = 545) |  | n | % Sample | % Emloyees in Germany | % Difference |
| Gender |  |  |  |  |  |
| Female |  | 262 | 48.4 | 46.8 | 1.7 |
| Male |  | 279 | 51.6 | 53.2 | -1.7 |
| Age |  |  |  |  |  |
| 20 years to under 35 years | | 136 | 25.9 | 28.8 | -2.9 |
| 35 years to under 50 years | | 200 | 38.1 | 36.1 | 2.0 |
| 50 years to unter 65 years | | 189 | 36.0 | 35.1 | 0.9 |
| Place of residence | |  |  |  |  |
| North |  | 72 | 13.3 | 17.7 | -4.4 |
| East |  | 106 | 19.6 | 17.1 | 2.5 |
| South |  | 178 | 32.9 | 30.7 | 2.2 |
| West |  | 185 | 34.2 | 34.5 | -0.3 |

Note. N = total sample size; n = frequency; % sample = percentage of valid responses in the present sample; % employees in Germany = percentage of employees in Germany, the the quotas for the percentage of employees in Germany were calculated using the population update for 2016 based on the 2011 census of the Federal Statistic Office (2018); Division of regions: North (Bremen, Hamburg, Mecklenburg-Western Pomerania, Lower Saxony, Schleswig-Holstein), East (Berlin, Brandenburg, Saxony, Saxony-Anhalt, Thuringia), South (Baden-Württemberg, Bavaria) and West (Hesse, North Rhine-Westphalia, Rhineland-Palatinate, Saarland).


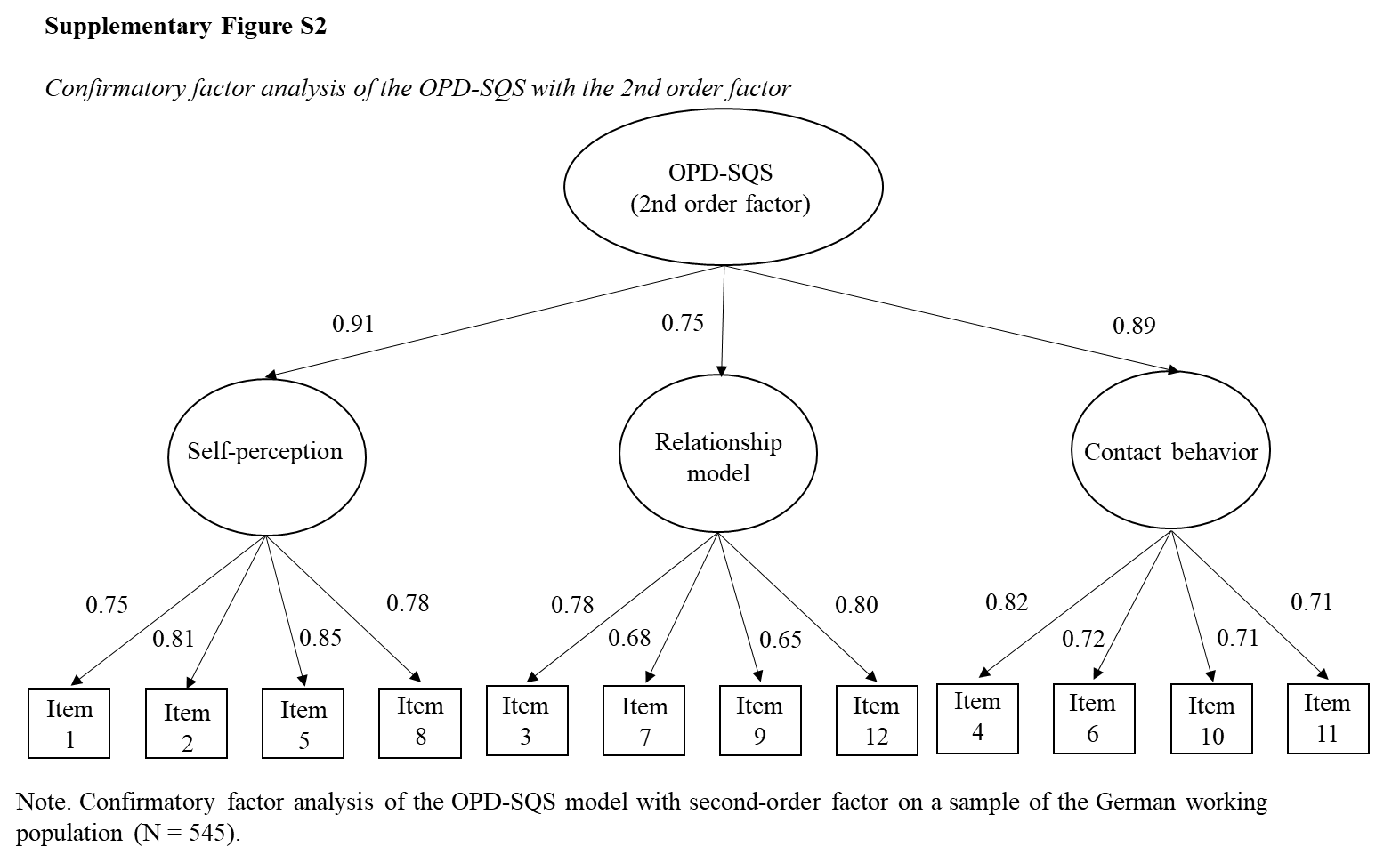


| **Supplementary Table S3** | | | | | | | |  |  |  |  |  |  |  |  |
| --- | --- | --- | --- | --- | --- | --- | --- | --- | --- | --- | --- | --- | --- | --- | --- |
| *Total, Direct and Indirect Effects* | | | | | | |  |  |  |  |  |  |  |  |  |
|  |  |  | BOSS-I | | |  |  |  |  | BOSS-II | | |  |  |  |
| Variable | Path | B | B SE | β | t | p | 95% CI | Path | B | B SE | β | t | p | 95% CI |  |
| K0 | a | -0.05 | 0.06 | -0.03 | -0.81 | .42 | [-0.18, 0.07] | a | -0.05 | 0.06 | -0.03 | -0.81 | .42 | [-0.18, 0.07] |  |
|  | b | **0.73** | **0.05** | **0.61** | **16.06** | **<.001** | [0.65, 0.82] | b | **0.86** | **0.05** | **0.63** | **17.27** | **<.001** | [0.76, 0.95] |  |
|  | c | -0.08 | 0.08 | -0.05 | -1.00 | .34 | [-0.24, 0.08] | c | -0.16 | 0.09 | -0.08 | -1.79 | .07 | [-0.34, 0.02] |  |
|  | c' | -0.04 | 0.07 | -0.02 | -0.58 | .56 | [-0.18, 0.10] | c' | -0.12 | 0.07 | -0.06 | -1.68 | .09 | [-0.26, 0.02] |  |
|  | ab | -0.04 |  |  |  |  | [-0.13, 0.05] | ab | 0.04 | 0.05 |  |  |  | [-0.15, 0.06] |  |
|  |  |  |  |  |  |  |  |  |  |  |  |  |  |  |  |
| K1a | a | **0.47** | **0.03** | **0.50** | **14.35** | **<.001** | [0.40, 0.53] | a | **0.47** | **0.03** | **0.50** | **14.35** | **<.001** | [0.40, 0.53] |  |
|  | b | **0.66** | **0.05** | **0.55** | **12.10** | **<.001** | [0.55, 0.77] | b | **0.80** | **0.06** | **0.63** | **13.43** | **<.001** | [0.68, 0.92] |  |
|  | c | **0.44** | **0.05** | **0.34** | **9.60** | **<.001** | [0.35, 0.53] | c | **0.47** | **0.05** | **0.39** | **9.53** | **<.001** | [0.38, 0.57] |  |
|  | c' | **0.14** | **0.05** | **0.12** | **2.65** | **.008** | [0.03, 0.24] | c' | 0.10 | 0.05 | 0.08 | 1.88 | .06 | [0.00, 0.21] |  |
|  | ab | 0.31 | 0.03 |  |  |  | [0.25, 0.38] | ab | 0.37 | 0.04 |  |  |  | [0.30, 0.45] |  |
|  |  |  |  |  |  |  |  |  |  |  |  |  |  |  |  |
| K1p | a | **0.48** | **0.04** | **0.43** | **11.34** | **<.001** | [0.39, 0.56] | a | **0.48** | **0.04** | **0.40** | **11.34** | **<.001** | [0.39, 0.56] |  |
|  | b | **0.64** | **0.06** | **0.53** | **11.60** | **<.001** | [0.53, 0.75] | b | **0.77** | **0.06** | **0.59** | **13.30** | **<.001** | [0.66, 0.89] |  |
|  | c | **0.56** | **0.06** | **0.42** | **9.83** | **<.001** | [0.45, 0.67] | c | **0.59** | **0.06** | **0.40** | **9.16** | **<.001** | [0.46, 0.72] |  |
|  | c' | **0.25** | **0.06** | **0.19** | **4.10** | **<.001** | [0.13, 0.37] | c' | **0.22** | **0.07** | **0.15** | **3.32** | **<.001** | [0.10, 0.35] |  |
|  | ab | 0.30 | 0.03 |  |  |  | [0.24, 0.38] | ab | 0.37 | 0.04 |  |  |  | [0.29, 0.46] |  |
|  |  |  |  |  |  |  |  |  |  |  |  |  |  |  |  |
| K2a | a | -0.03 | 0.07 | -0.02 | -0.44 | .66 | [0.17, 0.11] | a | -0.03 | 0.07 | -0.02 | -0.44 | .66 | [-0.17, 0.11] |  |
|  | b | **0.74** | **0.05** | **0.62** | **16.07** | **<.001** | [0.65, 0.83] | b | **0.86** | **0.05** | **0.66** | **17.42** | **<.001** | [0.76, 0.96] |  |
|  | c | **-0.08** | **0.09** | **-0.05** | **-0.95** | **.34** | [-0.25, 0.09] | c | -0.14 | 0.09 | -0.07 | -1.53 | .13 | [-0.32, 0.04] |  |
|  | c' | **-0.06** | **0.07** | **-0.03** | **-0.85** | **.40** | [-0.19, 0.08] | c' | -0.11 | 0.07 | 0.06 | -1.58 | .11 | [-0.25, 0.03] |  |
|  | ab | -0.02 | 0.05 |  |  |  | [-0.13, 0.08] | ab | -0.03 | 0.06 |  |  |  | [-0.15, 0.10] |  |
|  |  |  |  |  |  |  |  |  |  |  |  |  |  |  |  |
| K2p | a | **0.72** | **0.04** | **0.58** | **17.31** | **<.001** | [0.64, 0.80] | a | **0.72** | **0.04** | **0.58** | **17.31** | **<.001** | [0.64, 0.80] |  |
|  | b | **0.63** | **0.05** | **0.53** | **11.74** | **<.001** | [0.53, 0.74] | b | **0.80** | **0.06** | **0.61** | **13.66** | **<.001** | (0.69, 0.92] |  |
|  | c | **0.68** | **0.06** | **0.46** | **10.43** | **<.001** | [0.55, 0.81] | c | **0.70** | **0.07** | **0.43** | **10.05** | **<.001** | [0.57, 0.84] |  |
|  | c' | **0.22** | **0.07** | **0.15** | **3.11** | **.002** | [0.08, 0.36] | c' | 0.13 | 0.07 | 0.08 | 1.72 | .09 | [-0.02, 0.27] |  |
|  | ab | 0.46 | 0.05 |  |  |  | [0.37, 0.55] | ab | 0.57 | 0.05 |  |  |  | [0.48, 0.68] |  |
| Note. BOSS-I = burnout total score (job, self, family, and friends); BOSS-II = burnout total score (physical, cognitive, and emotional); CI = confidence interval; the values highlighted in grey do not represent satisfactory model quality regarding the verification of the one-dimensionality of the scale; K0 = repressed perception of conflict and emotions; K1a = individuation vs. dependency conflict in active mode; K1p = individuation vs. dependency conflict in passive mode; K2a = submission vs. control conflict in active mode; K2p = submission vs. control conflict in passive mode; significant effects (p < .05) are indicated in bold typing.  p < .05, p < 01, corrected p-value (Bonferroni) p < .001. | | | | | | | | | | | | | | | |
|  | | | | | | | |  |  |  |  |  |  |  |  |

| **Supplementary Table S3** | | | | | | |  |  | |  |  |  |  |  |  |
| --- | --- | --- | --- | --- | --- | --- | --- | --- | --- | --- | --- | --- | --- | --- | --- |
| *Total, Direct and Indirect effects* | | | | | |  |  |  | |  |  |  |  |  |  |
|  |  |  | BOSS-I | | |  |  | |  |  | BOSS-II | | |  |  |
| Variable | Path | B | B SE | β | t | p | 95% CI | | Path | B | B SE | β | t | p | 95% CI |
| K3a | a | **0.35** | **0.06** | **0.27** | **5.90** | **<.001** | [0.24, 0.47] | | a | **0.35** | **0.06** | **0.27** | **5.90** | **<.001** | [0.24, 0.47] |
|  | b | **0.74** | **0.05** | **0.62** | **15.52** | **<.001** | [0.64, 0.83] | | b | **0.85** | **0.05** | **0.65** | **16.61** | **<.001** | [0.75, 0.95] |
|  | c | **0.27** | **0.08** | **0.17** | **3.47** | **<.001** | [0.12, 0.43] | | c | **0.34** | **0.09** | **0.20** | **3.93** | **<.001** | [0.17, 0.51] |
|  | c' | 0.01 | 0.06 | 0.01 | 0.20 | .84 | [-0.11, 0.13] | | c' | 0.04 | 0.07 | 0.02 | 0.55 | .58 | [-0.10, 0.17] |
|  | ab | 0.26 | 0.05 |  |  |  | [0.17, 0.35] | | ab | 0.30 | 0.05 |  |  |  | [0.20, 0.41] |
|  |  |  |  |  |  |  |  | |  |  |  |  |  |  |  |
| K3p | a | 0.00 | 0.05 | 0.00 | 0.02 | .98 | [-0.10, 0.10] | | a | 0.00 | 0.05 | 0.00 | 0.02 | .98 | [-0.10, 0.10] |
|  | b | **0.74** | **0.05** | **0.62** | **16.05** | **<.001** | [0.65, 0.83] | | b | **0.86** | **0.05** | **0.66** | **17.34** | **<.001** | [0.76, 0.96] |
|  | c | -0.03 | 0.06 | -0.02 | -0.42 | .67 | [-0.15, 0.10] | | c | 0.01 | 0.07 | 0.01 | .021 | .84 | [-0.12, 0.15] |
|  | c' | -0.03 | 0.05 | -0.02 | -0.55 | .58 | [-0.13, 0.07] | | c' | 0.01 | 0.05 | 0.01 | 0.25 | .80 | [-0.09, 0.12] |
|  | ab | 0.01 | 0.04 |  |  |  | [-0.07, 0.08] | | ab | 0.00 | 0.04 |  |  |  | [-0.08, 0.09] |
|  |  |  |  |  |  |  |  | |  |  | . |  |  |  |  |
| K4a | a | **-0.33** | **0.05** | **-0.30** | **-6.51** | **<.001** | [-0.43, -0.23] | | a | **-0.33** | **0.05** | **-0.30** | **-6.51** | **<.001** | [-0.43, -0.23] |
|  | b | **0.73** | **0.05** | **0.07** | **15.32** | **<.001** | [0.63, 0.82] | | b | **0.82** | **0.05** | **0.63** | **16.10** | **<.001** | [0.72, 0.92] |
|  | c | **-0.28** | **0.06** | **-0.21** | **-4.36** | **<.001** | [-0.40, -0.15] | | c | **-0.42** | **0.07** | **-0.29** | **-6.26** | **<.001** | [-0.56, -0.30] |
|  | c' | -0.04 | 0.06 | -0.03 | -0.63 | .53 | [-0.15, 0.08] | | c' | **-0.15** | **0.06** | **-0.10** | **-2.54** | **.01** | [-0.27, -0.03] |
|  | ab | -0.24 | 0.04 |  |  |  | [-0.32, -0.17] | | ab | -0.27 | 0.05 |  |  |  | [-0.37, -0.19] |
|  |  |  |  |  |  |  |  | |  |  |  |  |  |  |  |
| K4p | a | **0.62** | **0.03** | **0.73** | **24.32** | **<.001** | [0.57, 0.67] | | a | **0.62** | **0.03** | **0.73** | **24.32** | **<.001** | [0.57, 0.67] |
|  | b | **0.32** | **0.07** | **0.27** | **4.94** | **<.001** | [0.19, 0.45] | | b | **0.38** | **0.07** | **0.27** | **5.57** | **<.001** | [0.25, 0.52] |
|  | c | **0.67** | **0.04** | **0.66** | **18.79** | **<.001** | [0.60, 0.74] | | c | **0.78** | **0.04** | **0.64** | **19.92** | **<.001** | [0.70, 0.85] |
|  | c' | **0.47** | **0.05** | **0.46** | **8.81** | **<.001** | [0.37, 0.58] | | c' | **0.54** | **0.06** | **0.32** | **9.36** | **<.001** | [0.42, 0.65] |
|  | ab | 0.20 | 0.04 |  |  |  | [0.12, 0.28] | | ab | 0.24 | 0.04 |  |  |  | [0.16, 0.32] |

Note. BOSS-I = burnout total score (job, self, family, and friends); BOSS-II = burnout total score (physical, cognitive, and emotional); Cl = confidence interval; K3a = need for care vs. self-sufficiency conflict in active mode; K3p = need for care vs. self-sufficiency conflict in passive mode; K4a = self-worth conflict in active mode; K4p = self-worth conflict in passive mode; significant effects (p <.05) are indicated by bold typing.

p < .05, p < 01, corrected p-value (Bonferroni) p < .001.

| **Supplementary Table S3** | | | | | | |  |  |  |  |  |  |  |  |
| --- | --- | --- | --- | --- | --- | --- | --- | --- | --- | --- | --- | --- | --- | --- |
| *Total, Direct and Indirect Effects* | | | | | | |  |  |  |  |  |  |  |  |
|  |  |  | BOSS-I | | |  |  |  |  | BOSS-II | | |  |  |
| Variable | Path | B | B SE | β | t | p | 95% CI | Path | B | B SE | β | T | p | 95% CI |
| K5a | a | **-0.31** | **0.06** | **-0.22** | **-5.34** | **<.001** | [-0.42, -0.20] | a | **-0.31** | **0.06** | **-0.22** | **-5.34** | **<.001** | [-0.42, -0.20] |
|  | b | **0.71** | **0.05** | **0.60** | **15.16** | **<.001** | [0.62, 0.81] | b | **0.83** | **0.05** | **0.63** | **16.40** | **<.001** | [0.73, 0.93] |
|  | c | **-0.36** | **0.08** | **-0.22** | **-4.55** | **<.001** | [-0.52, -0.21] | c | **-0.44** | **0.08** | **-0.24** | **-5.24** | **<.001** | [-0.60, -0.27] |
|  | c' | -0.14 | 0.07 | -0.08 | -2.15 | .03 | [-0.27, -0.01] | c' | **-0.18** | **0.07** | **0.10** | **-2.65** | **.01** | [-0.31, -0.05] |
|  | ab | -0.22 | 0.04 |  |  |  | [-0.31, -0.14,] | ab | -0.26 | 0.05 |  |  |  | [-0.36, -0.16] |
|  |  |  |  |  |  |  |  |  |  |  |  |  |  |  |
| K5p | a | **0.53** | **0.04** | **0.53** | **14.55** | **<.001** | [0.46, 0.60] | a | **0.53** | **0.04** | **0.53** | **14.55** | **<.001** | [0.46, 0.60] |
|  | b | **0.61** | **0.05** | **0.51** | **11.61** | **<.001** | [0.51, 0.71] | b | **0.77** | **0.06** | **0.59** | **13.09** | **<.001** | [0.66, 0.89] |
|  | c | **0.55** | **0.05** | **0.47** | **10.34** | **<.001** | [0.45, 0.66] | c | **0.57** | **0.06** | **0.43** | **9.43** | **<.001** | [0.45, 0.69] |
|  | c' | **0.23** | **0.06** | **0.20** | **4.22** | **<.001** | [0.12, 0.34] | c' | **0.16** | **0.06** | **0.12** | **2.63** | **.01** | [0.04, 0.28] |
|  | ab | 0.32 | 0.03 |  |  |  | [0.26, 0.39] | ab | 0.41 | 0.04 |  |  |  | [0.33, 0.48] |
|  |  |  |  |  |  |  |  |  |  |  |  |  |  |  |
| K6a | a | 0.09 | 0.55 | 0.07 | 1.59 | .11 | [-0.02, 0.20] | a | 0.09 | 0.06 | 0.07 | 1.59 | 0.11 | [-0.02, 0.20] |
|  | b | **0.73** | **0.05** | **0.61** | **15.68** | **<.001** | [0.64, 0.82] | b | **0.86** | **0.05** | **0.65** | **17.08** | **<.001** | [0.76, 0.95] |
|  | c | **0.18** | **0.07** | **0.12** | **2.65** | **.008** | [0.05, 0.32] | c | **0.15** | **0.08** | **0.10** | **2.02** | **.04** | [0.01, 0.30] |
|  | c' | 0.12 | 0.06 | 0.08 | 1.93 | .05 | [0.02, 0.24] | c' | 0.08 | 0.06 | 0.05 | 1.30 | .20 | [-0.04, 0.19] |
|  | ab | 0.06 | 0.04 |  |  |  | [-0.01, 0.14] | ab | 0.08 | 0.05 |  |  |  | [-0.02, 0.17] |
|  |  |  |  |  |  |  |  |  |  |  |  |  |  |  |
| K6p | a | **0.45** | **0.04** | **0.43** | **10.60** | **<.001** | [0.37, 0.54] | a | **0.45** | **0.04** | **0.43** | **10.60** | **<.001** | [0.37, 0.54] |
|  | b | **0.73** | **0.05** | **0.61** | **14.33** | **<.001** | [0.63, 0.83] | b | **0.86** | **0.05** | **0.66** | **16.23** | **<.001** | [0.76, 0.97] |
|  | c | **0.35** | **0.06** | **0.28** | **6.13** | **<.001** | [0.24, 0.46] | c | **0.38** | **0.06** | **0.28** | **6.40** | **<.001** | [0.27, 0.50] |
|  | c' | 0.02 | 0.06 | 0.01 | 0.32 | .75 | [-0.09, 0.13] | c' | -0.01 | 0.05 | 0.01 | -0.13 | <.90 | [-0.11, 0.10] |
|  | ab | 0.33 | 0.04 |  |  |  | [0.25, 0.41] | ab | 0.39 | 0.05 |  |  |  | [0.30, 0.48] |

Note. BOSS-I = burnout total score; (job, self, family, and friends); BOSS-II = burnout total score (physical, cognitive, and emotional); Cl = confidence interval; K5a = guilt conflict in active mode; K5p = guilt conflict in passive mode; K6a = oedipal conflict in active mode; K6p = oedipal conflict in passive mode; significant effects (p < .05) are indicated by bold typing.

p < .05, p < 01, corrected p-value (Bonferroni) p < .001.
